# Higher serum betatrophin level in type 2 diabetes subjects is associated with urinary albumin excretion and renal function

**DOI:** 10.1186/s12933-015-0326-9

**Published:** 2016-01-07

**Authors:** Chang-Chiang Chen, Hendra Susanto, Wen-Han Chuang, Ta-Yu Liu, Chih-Hong Wang

**Affiliations:** Department of Biological Science and Technology, National Chiao Tung University, 75 Bo-Ai Street, Hsinchu, 300 Taiwan; Department of Internal Medicine, National Taiwan University Hospital Hsin-Chu Branch, Hsinchu, 300 Taiwan

**Keywords:** Betatrophin, Type 2 diabetes, Diabetic nephropathy, ACR, eGFR

## Abstract

**Background:**

Betatrophin is a newly identified liver-derived hormone that is associated with glucose homeostasis and lipid metabolism. Although dysregulated lipid metabolism results in diabetic nephropathy (DN) development in patients with type 2 diabetes mellitus (T2DM), it is not understood whether betatrophin is associated with urinary albumin excretion and renal function.

**Methods:**

Based on albumin/creatinine ratio (ACR), 109 T2DM patients were divided into normoalbuminuria (ACR <30 mg/g), microalbuminuria (ACR between 30 and 300 mg/g), and macroalbuminuria (ACR > 300 mg/g). Serum betatrophin levels of 109 T2DM patients and 32 healthy subjects were determined by enzyme-linked immunosorbent assay (ELISA).

**Results:**

Serum level of betatrophin was significantly increased in T2DM patients with normoalbuminuria, microalbuminuria, and macroalbuminuria as compared with healthy subjects (*P* < 0.001). Serum betatrophin level was positively correlated with sex, duration of diabetes, systolic blood pressure (SBP), body mass index (BMI), ACR, and triglyceride, whereas it was inversely correlated with estimated glomerular filtration rate (eGFR), total cholesterol, and high-density lipoprotein cholesterol (HDL-C) (*P* < 0.001). Furthermore, multivariate regression analysis showed the betatrophin was significantly and positively independent with triglyceride and low-density lipoprotein cholesterol (LDL-C) (*P* < 0.05), whereas it was inversely independent with eGFR, total cholesterol, and low-density lipoprotein cholesterol (HDL-C) (*P* < 0.05). In addition, the betatrophin had higher odds of having DN [odds ratio (OR) = 5.65, 95 % confidence interval (CI) 2.17–14.57, *P* < 0.001].

**Conclusion:**

Betatrophin is significantly increased in T2DM patients with different stages of albuminuria. Betatrophin may be a novel endocrine regulator involved in DN development.

## Background

Type 2 diabetes mellitus (T2DM) is one of the most severe public health problems and affects over 170 million people worldwide [1]. The major pathogenesis of T2DM is insulin resistance, which is the resistance of target tissues to insulin action, and has been suggested to play a key role in the cluster of diabetic complications including atherosclerosis, and diabetic nephropathy (DN) [2, 3]. Recent studies in human autopsy and in a mouse model of induced insulin resistance have also suggested that patients with T2DM cause insulin resistance due to a decrease in β-cell mass by apoptosis [4]. Insulin stimulates albumin gene expression and albumin secretion from hepatocyte in both healthy subjects and diabetic patients. Reduced serum albumin levels are observed in DN, whereas high serum albumin levels have been reported to be associated with metabolic syndrome, an indicator of obesity and overnutrition [5]. In addition, recently, serum albumin has been suggested to be associated with insulin resistance. The failure of β-cells compensates for ambient insulin resistance that leads to uncontrolled hyperglycemia resulted in renal glomerular hyperfiltration with microalbuminuria [6]. However, the relationship between β-cells and albuminuria or renal functions in T2DM patients remains unclear.

Betatrophin is a newly recognized liver-derived hormone that has been implicated in both glucose and lipid metabolism [7–9]. Recent study has pointed out that mice treated S961 caused insulin resistance resulting in β-cell proliferation via overexpressing betatrophin [10]. Moreover, serum level of betatrophin is positively associated with type 1 Diabetes mellitus (T1DM) and T2DM [9, 11, 12], hyperlipidemia [13], and indexes of insulin resistance [14]. However, increased levels of betatrophin in T1DM is in contradiction with the initial finding in an insulin-deficient mouse model by Meltons group [11] and there are mixed data regarding betatrophin levels in T2DM. A number of studies reported that betatrophin was increased in T2DM patients [4, 8, 12], while Gomez-Ambrosi et al. studies showed that circulating betatrophin level was reduced in T2DM patients [15]. In addition, mice lacking betatrophin showed a reduction in plasma triglyceride levels in response to refeeding, whereas hepatic overexpression of betatrophin caused hypertriglyceridemia without changing glucose metabolism [7]. The circulating concentrations of betatrophin are also significantly correlated with atherogenic lipid profiles in high-risk cohorts with T2DM or cardiovascular disease [14]. More recently, Ebert showed that betatrophin levels are correlated with clinical renal function [16]. Nevertheless, hyperglycemia and altered lipid profile are associated with DN development. It is not clearly understood whether betatrophin levels are correlated with albuminuria in T2DM.

The aim of this study was to investigate circulating betatrophin concentrations in healthy subjects and type 2 diabetic patients with albuminuria, and to determine whether betatrophin levels are associated with DN development. We hypothesized that betatrophin concentrations are correlated with albuminuria and associated with renal function.

## Methods

### Study population

The study protocol was approved by the Institutional Review Board of the National Taiwan University Hospital Hsin-Chu Branch, Hsinchu, Taiwan (No: 104-001-E). The written informed consents were obtained from all participants and all studies were carried out in accordance with the approved guideline. Participants were enrolled between February 2014 and January 2015. All subjects were investigated in the morning after an overnight fast. A total of 141 Taiwanese subjects were enrolled, of which 32 were healthy subjects and 109 type 2 diabetes mellitus. Among the type 2 diabetic patients, 37 patients had normoalbuminuria (ACR < 30 mg/g), 37 had microalbuminuria (ACR between 30 and 300 mg/g), and 35 had macroalbuminuria (ACR > 300 mg/g). The healthy subjects were selected as subjects without disease and not taking any medications. Exclusion criteria were as follows: (1) any evidence of active infection (e.g. fever, or leukocytosis); (2) any evidence of impaired renal, hepatic, or hematopoietic function; (3) no known history of chronic systemic diseases, such as diabetes and hypertension; (4) no long-term medical treatment for chronic systemic diseases; (5) blood tests showing abnormal glucose levels.

### Major metabolic indicators

Blood samples taken after an overnight fast were kept in an icebox immediately after collection, and the serum was separated from erythrocytes by centrifugation at 1500×*g* for 10 min at 4 °C. The serum, if not analyzed, was frozen at minus 80 °C within 30 min of collection. The estimated glomerular filtration rate (eGFR) was calculated using the simplified modification of diet in renal disease (MDRD) study equation.

### Laboratory analysis

Blood samples were collected after overnight fasting, and serum and urine were stored at minus 20 °C. Serum variables were analyzed at the Department of Medical and Chemical Laboratory Diagnostics at the National Taiwan University Hospital Hsin-Chu Branch by using routine procedures. Serum and urine levels of betatrophin were quantified using a commercially available ELISA kit (Wuhan Eiaab Science, Wuhan, China; catalogue No. E11644h) according to the manufacturer’s instructions [17]. Current ELISA kit was validated against other available kits showing correlation coefficient of 0.992. The C-terminal fragment of betatrophin was quantified using different human betatrophin ELISA kit (Phoenix EK-051-55).

### Statistical analysis

All statistical analyses were performed using SPSS Software version 21.0 (Chicago, IL, USA). Differences in circulating level of betatrophin in healthy subjects and T2DM patients with different stages of albuminuria were assessed by parametric one-way analysis of variance (ANOVA) with Turkey post hoc test. Univariate correlations were performed using non-parametric Spearman’s rank correlation method. Afterward, multivariate linear regression analysis was performed to identify independent relationships. Before multivariate correlation analyses were calculated, distribution of the respective variables was tested for normality using Kolmogorof-Smirnov test and normally distributed parameters were logarithmically transformed. A P value less than 0.05 was considered statistically significant.

## Results

### Baseline characteristics of the total sample

Table 1 summarizes the clinical characteristics of the 4 groups including healthy subjects, and T2DM patients with normoalbuminuria, microalbuminuria and macroalbuminuria. The data revealed that age, duration of DM, systolic blood pressure (SBP), body mass index (BMI), fasting blood glucose (FBG), albumin to creatinine ratio (ACR), hemoglobin A1c (HbA1c), high-sensitivity C-reactive protein (hs-CRP), triglycerides, and ACR in T2DM patients with albuminuria had a significant increase than in health subjects, whereas eGFR had a markedly decrease in T2DM patients with albuminuria than in health subjects. There was no statistically significant difference between healthy subjects and T2DM patients with albuminuria in low-density lipoprotein cholesterol (LDL-C), and total cholesterol levels.

**Table 1:** Subject characteristics and metabolic parameters

	Healthy	Normoalbuminuria	Microalbuminuria	Macroalbuminuria
N	32	37	37	35
Duration of diabetes (years)	–	7.6 ± 1.1*	10.6 ± 1.1*^†^	12.0 ± 1.5*^†,‡^
Age	50.8 ± 1.2	56.2 ± 1.1*	57.2 ± 0.8*	55.9 ± 1.2*
SBP (mmHg)	100.4 ± 7.3	125.3 ± 4.2*	134.1 ± 3.1*	138.1 ± 2.5*
BMI (kg/m^2^)	22.7 ± 0.9	25.6 ± 0.5*	26.9 ± 0.5*	27.0 ± 0.8*
Hyperlipidemia (% Y)	0	62.16	72.97	74.28
Hypertension (%)	0	54.05	78.39	82.86
Smoking (% Y)	0	13.51	21.62	25.71
Alcoholism (% Y)	0	13.51	16.21	0
High protein diet (% Y)	0	21.62	24.32	14.28
FBG (mg/dL)	90.97 ± 1.48	142.24 ± 7.09*	157.11 ± 7.10*	153.51 ± 10.04*
eGFR (mL/min/1.73 m^2^)	93.50 ± 3.07	85.09 ± 3.35	79.89 ± 4.87*	47.65 ± 5.10*^,†^
ACR (mg/g)	5.7 ± 0.4	12.8 ± 1.2*	100.5 ± 12.4*^,†^	2229.6 ± 376*^,†,‡^
HbA1c (%)	5.8 ± 0.1	7.8 ± 0.2*	8.3 ± 0.3*	8.5 ± 0.4*
hsCRP (mg/dL)	0.13 ± 0.01	0.29 ± 0.09*	0.22 ± 0.04*	0.67 ± 0.18*^,†,‡^
Triglyceride (mg/dL)	118.5 ± 12.1	117.7 ± 14.2	146.5 ± 17.2*	197.6 ± 28.4*^,†,‡^
LDL cholesterol (mg/dL)	119.1 ± 4.8	109.3 ± 5.9	113.6 ± 5.6	125.5 ± 8.4
HDL cholesterol (mg/dL)	54.9 ± 2.9	55.0 ± 2.3	53.2 ± 3.2*	44.7 ± 1.9*^,†,‡^

Data are mean ± SEM

*SBP* systolic blood pressure, *FBG* fasting blood glucose, *ACR* urine albumin/creatinine ratio, *% Y* % of positive patients with those risk factors of DN

* P < 0.05 vs. healthy subjects, ^†^P < 0.05 vs. normoalbuminuria, ^‡^ P < 0.05 vs. microalbuminuria

### Elevated serum level of betatrophin in T2DM patients with albuminuria

**Fig. 1:**
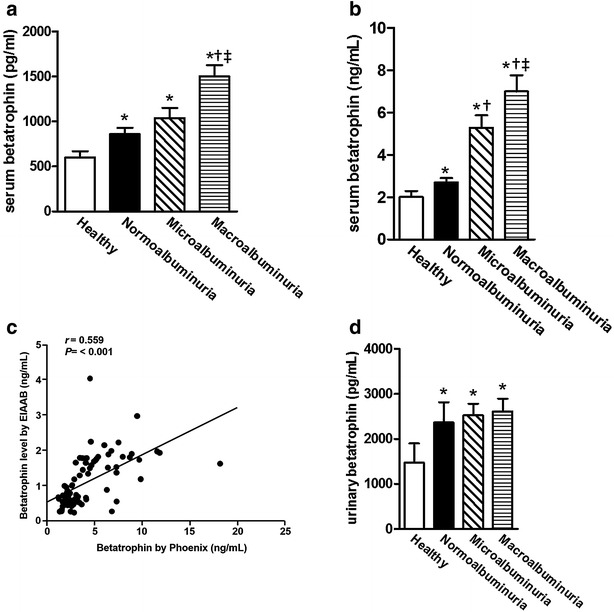
Serum and urine betatrophin concentrations of healthy and T2DM patients with normoalbuminuria, microalbuminuria and macroalbuminuria. **a** Serum full-length betatrophin level was measured by N-terminal kit and **b** serum total betatrophin level was measured by C-terminal kit. **c** Correlations between N-terminal kit and C-terminal kit. **d** Urinary betatrophin level was measured by N-terminal kit. Data are mean ± SEM. *P < 0.05 vs. healthy subjects; ^†^P < 0.05 vs. normoalbuminuria; ^‡^P < 0.05 vs. microalbuminuria

Previous study has shown that betatrophin is correlated with renal function [16] and both albumin and betatrophin are produced by liver. We also investigated whether circulating serum level of betatrophin is associated with T2DM patients with albuminuria. We found that serum full-length and total betatrophin levels were significantly increased in T2DM patients with normoalbuminuria, microalbuminuria, and macroalbuminuria (*P* < 0.001; Fig. 1a, b) compared with healthy subjects. Both full-length and total betatrophin concentrations were determined in serum samples by both N-terminal and C-terminal kits. Both ELISA kits correlated significantly with one another (*r* = 0.559; *P* < 0.001; Fig. 1c). We also performed betatrophin level in urine samples. Although urinary betatrophin level was significantly increased in T2DM patients as compared with healthy subjects, it was no differences among normoalbuminuria, microalbuminuria, and macroalbuminuria groups (Fig. 1d). Thus, we rule out that reduced clearance of betatrophin results in decreased glomerular filtration in the different groups. Taken together, the data indicates circulating level of betatrophin is correlated with T2DM patients with different stages of albuminuria and loss of albumin may result in increased betatrophin produce.

### Univariate correlations of betatrophin in total sample

To investigate whether betatrophin is correlated with metabolic parameters, we used Spearman’s correlation. As shown by Spearman’s correlation, serum betatrophin level in all individuals was positively and significantly correlated with duration of DM (*r* = 0.385, *P* < 0.001), SBP (*r* = 0.194, *P* = 0.032), BMI (*r* = 0.194, *P* = 0.031), FBG (*r* = 0.175, *P* = 0.048), ACR (*r* = 0.427, *P* < 0.001), HbA1_C_ (%) (*r* = 0.264, *P* = 0.003), and triglycerides (*r* = 0.282, *P* = 0.001) (Table 2). In contrast, betatrophin was significantly and inversely correlated with sex (*r* = −0.331 for male, *P* < 0.001), eGFR (*r* = −0.454, *P* < 0.001), total cholesterol (*r* = −0.216, *P* = 0.018) and high-density lipoprotein cholesterol (HDL-C) (*r* = −0.391, *P* < 0.001) (Table 2). After correlation for each of the individual groups, we found that serum betatrophin level was positively correlated with FBG (*r* = 0.357; *P* = 0.045 in microalbuminuria group), ACR (*r* = 0.342; *P* = 0.042 in normoalbuminuria and *r* = 0.343; *P* = 0.049 in microalbuminuria groups), HBA1_C_ (*r* = 0.361; *P* = 0.045 in microalbuminuria group) and triglyceride (*r* = 0.368; *P* = 0.042 in microalbuminuria group). Its level was inversely correlated with age (*r* = −0.362; *P* = 0.033 in macroalbuminuria group), sex (*r* = −0.485; *P* = 0.008 in healthy group, and *r* = −0.530; *P* = 0.003 in microalbuminuria group), eGFR (*r* = −0.375; *P* = 0.024 in normoalbuminuria group, and *r* = −0.435; *P* = 0.018 in microalbuminuria group), total cholesterol (*r* = −0.434; *P* = 0.019 in microalbuminuria group), and HDL-cholesterol (*r* = −0.593; *P* < 0.001 in microalbuminuria group, and *r* = −0.446; *P* = 0.007 in macroalbuminuria group).

**Table 2:** Univariate correlations of parameters with serum betatrophin level in all participants, and T2DM patients with normoalbuminuria, microalbuminuria, and macroalbuminuria

Parameters	All participants *r* (*P* value)	Healthy *r* (*P* value)	Normoalbuminuria *r* (*P* value)	Microalbuminuria *r* (*P* value)	Macroalbuminuria *r* (*P* value)
Age (years)	0.131 (0.145)	−0.108 (0.960)	−0.123 (0.476)	0.278 (0.145)	−0.362 (0.033*)
Sex (Male/Female)	−0.331 (<0.001*)	−0.485 (0.008*)	0.086 (0.625)	−0.530 (0.003*)	−0.278 (0.105)
DM duration (years)	0.385 (<0.001*)	–	0.183 (0.292)	−0.392 (0.035*)	0.009 (0.960)
SBP (mmHg)	0.194 (0.032*)	0.113 (0.583)	0.040 (0.821)	0.104 (0.592)	−0.166 (0.342)
DBP (mmHg)	0.141 (0.122)	−0.012 (0.955)	0.167 (0.346)	0.281 (0.140)	−0.324 (0.057)
BMI (kg/m^2^)	0.194 (0.031*)	0.010 (0.961)	0.105 (0.550)	0.025 (0.897)	0.192 (0.269)
FBG (mg/dL)	0.175 (0.048*)	−0.002 (0.993)	−0.040 (0.819)	0.357 (0.045*)	−0.076 (0.663)
ACR (mg/g)	0.427 (<0.001*)	−0.180 (0.359)	0.342 (0.042*)	0.343 (0.049*)	0.025 (0.890)
eGFR (ml/min/1.73 m^2^)	−0.454 (<0.001*)	−0.108 (0.578)	−0.375 (0.024*)	−0.435 (0.018*)	−0.239 (0.166)
HbA1c (%)	0.264 (0.003*)	0.169 (0.380)	0.289 (0.077)	0.361 (0.045*)	−0.127 (0.469)
hsCRP (mg/dL)	0.133 (0.138)	−0.108 (0.578)	0.009 (0.959)	−0.208 (0.278)	−0.135 (0.438)
Triglyceride (mg/dL)	0.282 (0.001*)	0.184 (0.338)	−0.222 (0.200)	0.368 (0.042*)	0.224 (0.196)
Total Cholesterol (mg/dL)	−0.261 (0.001*)	0.102 (0.599)	0.176 (0.313)	−0.434 (0.019*)	−0.192 (0.270)
LDL cholesterol (mg/dL)	0.017 (0.853)	0.027 (0.889)	0.236 (0.172)	−0.278 (0.144)	−0.027 (0.880)
HDL cholesterol (md/dL)	−0.391 (<0.001*)	−0.293 (0.130)	0.132 (0.449)	−0.593 (<0.001*)	−0.446 (0.007*)

*SBP* systolic blood pressure, *DBP* diastolic blood pressure, *BMI* body mass index, *FBG* fasting blood glucose, *ACR* albumin /creatinine ratio, *eGFR* estimated glomerular filtration rate, *HbA1c* hemoglobin A1c, *hsCRP* high sensitive C-reactive protein, *r* coefficient correlation

* P value <0.05 significant correlation as assessed by Spearman’s correlation method

### Multivariate regression analysis in the total sample

To verify independent associations, multiple linear regression analysis was performed. The eGFR showed an independent and significant predictor of betatrophin (*P* < 0.05; Table 3). Furthermore, circulating level of betatrophin was significantly and positively independent with triglyceride, and LDL-C (*P* < 0.05), whereas it was inversely independent with total cholesterol (*P* < 0.05; Table 3).

**Table 3:** Multivariate regression analysis with betatrophin as dependent variable

Independent variables	β (95 % CI)	*P*
Age	−0.008 (−0.025 to 0.008)	0.324
Sex	−0.207 (−0.436 to 0.022)	0.076
Duration of diabetes	0.003 (−0.014 to 0.019)	0.748
SBP	0.002 (−0.005 to 0.008)	0.596
BMI	−0.002 (−0.032 to 0.027)	0.888
FBG	0.000 (−0.003 to 0.003)	0.915
eGFR (ml/min/1.73 m^2^)	−0.005 (−0.010 to 0.001)	0.039
ACR	6.875 (0.000 to 0.000)	0.443
HbA1c (%)	0.038 (−0.040 to 0.116)	0.338
hsCRP	−0.063 (−0.205 to 0.079)	0.383
Triglyceride	0.001 (0.000 to 0.003)	0.036*
LDL cholesterol	0.005 (0.000 to 0.009)	0.049*
HDL cholesterol	0.001 (−0.008 to 0.010)	0.832
Total cholesterol	−0.005 (−0.010 to 0.000)	0.026*

Multivariate regression analysis between betatrophin (dependent variable) and independent variables shown

Independent variables

Standardized β-coefficients and P value are given

* *P* < 0.05 is significant correlation

### Betatrophin versus DN risk factors

**Fig. 2:**
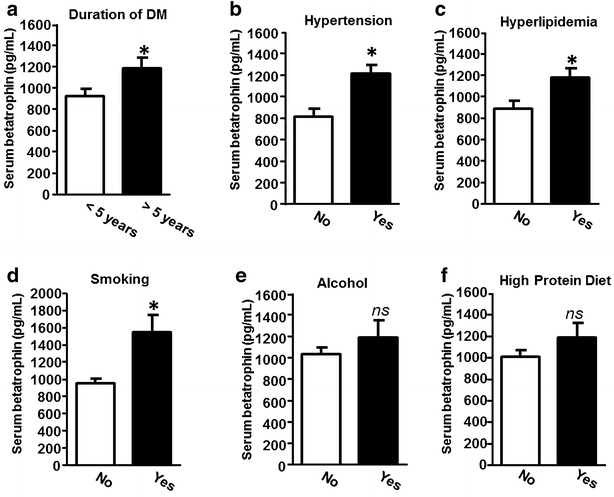
Serum level of betatrophin is associated with the risk factor of DN. **a** Duration of DM, **b** hypertension, **c** hyperlipidemia, **d** smoking, **e** alcohol, and **f** high protein diet. Data are shown as mean ± SEM. *P < 0.05 and *ns* not significant

DN risks including duration of DM, hypertension, hyperlipidemia, smoking, and high protein diet have been addressed. We next investigated whether betatrophin is associated with duration of DM, hypertension, hyperlipidemia, smoking, and high protein diet of T2D with albuminuria. The data showed that the serum betatrophin level was significantly increased in the type 2 diabetic patients with >5 years duration of DM, hypertension, hyperlipidemia, and smoking, but there was no significant difference of betatrophin level in patients who consumed alcohol or had a high protein diet (Fig. 2).

### Betatrophin versus nephropathy

When the betatrophin was related to DN (ACR > 300 mg/g) in a multivariate binary logistic regression model, serum level of betatrophin was significantly related to DN [odds ratio (OR) 5.63 (95 % CI 2.17–14.57), *P* < 0.001] (Table 4) without adjustment. Following multiple adjustment with Age, sex, and BMI, we observed that the OR for hsCRP was significantly related to DN [2.73 (95 % CI 1.11–6.72), *P* = 0.029], while betatrophin had the highest OR and was significantly related to DN [OR 5.65 (95 % CI 2.17–14.57), *P* < 0.001] (Table 4). This result suggests that betatrophin may serve as predictor of DN.

**Table 4:** OR (95 % CI) by binary logistic regression models for nephropathy with ACR as dependent variable

Models	Covariates	ACR (n = 141)			
		Normal (n = 106)		Nephropathy (n = 35)	
		OR (95 % CI)	P	OR (95 % CI)	P
Model 1	Betatrophin	1.00	–	5.63 (2.17–14.57)	<0.001*
	DM duration	1.00	–	1.08 (1.00–1.15)	0.040*
	hsCRP	1.00	–	1.91 (0.91–3.98)	0.086
	SBP	1.00	–	1.03 (0.99–1.06)	0.103
	HbA1c	1.00	–	1.11 (0.86–1.44)	0.434
	TG	1.00	–	1.00 (0.99–1.01)	0.092
Model 2	Betatrophin	1.00	–	6.36 (2.78–14.55)	<0.001*
	DM duration	1.00	–	1.09 (1.03–1.16)	0.002*
	hsCRP	1.00	–	2.90 (1.17–7.20)	0.022*
	SBP	1.00	–	1.03 (1.01–1.06)	0.012*
	HbA1c	1.00	–	1.27 (1.03–1.56)	0.026*
	TG	1.00	–	1.01 (1.00–1.01)	0.005*
Model 3	Betatrophin	1.00	–	5.79 (2.51–13.32)	<0.001*
	DM duration	1.00	–	1.10 (1.05–1.17)	<0.001*
	hsCRP	1.00	–	3.19 (1.28–7.97)	0.013*
	SBP	1.00	–	1.04 (1.01–1.06)	0.004*
	HbA1c	1.00	–	1.33 (1.08–1.64)	0.007*
	TG	1.00	–	1.01 (1.00–1.01)	0.006*
Model 4	Betatrophin	1.00	–	5.43 (2.44–12.09)	<0.001*
	DM duration	1.00	–	1.10 (1.04–1.16)	0.001*
	hsCRP	1.00	–	2.44 (1.10–5.43)	0.029*
	SBP	1.00	–	1.03 (1.01–1.06)	0.010*
	HbA1c	1.00	–	1.28 (1.04–1.56)	0.018*
	TG	1.00	–	1.00 (1.00–1.01)	0.014*
Model 5	Betatrophin	1.00	–	5.65 (2.39–13.33)	<0.001*
	DM duration	1.00	–	1.10 (1.03–1.16)	0.002*
	hsCRP	1.00	–	2.73 (1.11–6.72)	0.029*
	SBP	1.00	–	1.03 (1.00–1.06)	0.021*
	HbA1c	1.00	–	1.26 (1.01–1.56)	0.036*
	TG	1.00	–	1.01 (1.00–1.01)	0.017*

*Model 1* unadjusted, *Model 2* age adjusted, *Model 3* sex adjusted, *Model 4* BMI adjusted, *Model 5* age, sex, and BMI adjusted, *hsCRP* high sensitive C-reactive protein, *SBP* systolic blood pressure, *HbA1c* hemoglobin A1c, *TG* triglycerides

* P < 0.05 is significant in all model

## Discussion

In this study, we show that serum level of betatrophin was significantly increased in T2DM patients with normoalbuminuria, microalbuminuria, and macroalbuminuria compared with healthy subjects (*P* < 0.001), in particular macroalbuminuric type 2 diabetic patients. Furthermore, serum level of betatrophin was positively correlated with duration of DM, SBP, BMI, FBG, HbA1_C_, ACR, and triglyceride, whereas it was inversely correlated with eGFR, and HDL-C (*P* < 0.001). However, after correlation for each of the individual groups, we found that serum betatrophin level was positively correlated with FBG, ACR, HBA1_C_, and triglyceride, while its level was inversely correlated with sex, eGFR, total cholesterol, and HDL-cholesterol in microalbuminuria group. Importantly, we found that betatrophin had higher odds of having DN progression. Thus, betatrophin may be a novel endocrine regulator involved in DN development.

### Increased serum betatrophin level in T2DM patients

Betatrophin has been suggested a liver-derived hormone that is capable of inducing β-cell proliferation by Yi et al. [10], and a number of studies showed that betatrophin upregulated in T2DM patients [12, 18, 19], while Gomez-Ambrosi et al. studies showed that circulating betatrophin level was reduced in T2DM patients [15]. In addition, loss of albumin in urine has been implicated insulin resistance, which causes liver produced hormones as potential mediators of the increased β-cell proliferation in T2DM [6, 20]. However, it is still unknown whether loss of albumin in urine causes increased serum betatrophin level or not. In the current study, our data in agreement with previous studies that serum level of betatrophin was increased in T2DM patients as compared with healthy subjects, especially in macroalbuminuria group. This increase may be related to loss of albumin causing increased insulin resistance and higher demand for insulin in T2DM patients with albuminuria. Importantly, we found a significant association between betatrophin and glucose or HbA1c. Recent studies have shown that betatrophin levels was up-regulated by 3–4 folds at the transcription level in the liver of the *db/db a*nd *ob/ob* mice models as compared with wild-type mice [10]. This trend in increased betatrophin level was also observed in patients with longer duration of T2DM in our population [4, 8]. However, there are mixed data regarding betatrophin level in T2DM studies. A recent report found that betatrophin level was reduced in T2DM patients [15]. Fu et al. suggested that the discrepancies were caused by different sample size, BMI, and ethnic groups in T2DM [21]. Therefore, role of betatrophin in T2DM is need to elucidate in future studies.

### Effect of age and BMI on the betatrophin level

There are several factors affect circulating betatrophin level including age and BMI. Abu-Farha M et al. studies have reported that betatrophin level was not associated with T2DM [22, 23] and age may affect this results. In the present study, we observed that serum betatrophin level was inversely correlated with age in T2DM patients with macroalbuminuria group. Age has related to mitochondria dysfunction, increased oxidative stress, inflammation, and hormonal changes [24], which play an important role in the pathogenesis of DN progression. Moreover, we found that BMI was significantly increased in T2DM patients as compared with healthy subjects. In agreement with our findings, increased expression of the betatrophin has been shown in T2DM patients with higher BMI [12], and in obese individuals [25]. Ebert et al. have also demonstrated that insulin is a direct dose-and time-dependent to induce betatrophin mRNA expression in differentiated 3T3-L1 adipocytes in vitro [16]. Thus, fat tissue and its adipokines may affect betatrophin expression and secretion from hepatocytes. Thus, we can not rule out the factors age, and BMI resulting in increased circulating betatrophin level in T2DM patients with albuminuria in our study.

### Serum lipids is correlated with betatrophin and potentially contributes to progression of DN

It is known that dyslipidemia potentially contributes to progression of DN [26, 27]. Recent studies have confirmed a link between serum lipids and DN [28]. Importantly, several studies reported that triglycerides and cholesterol have different effects on the progression of nephropathy, depending upon the duration of diabetes [29, 30]. Accumulation of lipids in kidney causes increased advanced glycation end-products (AGEs), inflammatory cytokines, and reactive oxygen species (ROS) resulted in endothelial dysfunction, glomerulosclerosis and tubulointerstitial injury in T2DM [31–33]. In this study, the multivariate regression analysis showed serum betatrophin level was a positive and independent predictor of DN. Its level was significantly and positively independent with triglyceride (*P* < 0.05), whereas it was inversely independent with eGFR, total cholesterol, and HDL-C (*P* < 0.05). DN has been characterized by persistent albuminuria (>300 mg/g) and progressive decline in the eGFR. DN causes albumin loss in urine resulted in reduced serum albumin. Serum albumin levels have been associated with lipid metabolism. The primary role of albumin is to transport fatty acids to liver. Betatrophin is known to play a key role in lipid metabolism [14, 34] and our study show that serum betatrophin level was significantly associated with triglyceride and LDL-C (*P* < 0.05). In agreement with our findings, mice lacking betatrophin had a 70 % reduction in plasma levels of triglycerides compared to wild-type control mice [7], whereas adenovirus-mediated hepatic overexpression of betatrophin increased plasma triglyceride levels more than fivefold [35]. Moreover, Fenzl et al. found that serum betatrophin level was associated with triglycerides, HDL-C, and apolipoprotein B in type 2 diabetic patients [14]. Thus, dysregulated lipid metabolism may be the potential mechanism involved betatrophin in the development of DN. Moreover, we also observed betatrophin was inversely correlated with eGFR, whereas previous studies showed serum level of betatrophin has a positive associated with eGFR in T2DM patients with hemodialysis [16]. The disparities may be simply due to the samples from chronic kidney disease with hemodialysis, not T2DM with albuminuria. Hemodialysis is a process that uses a man-made membrane to remove wastes, such as urea and albumin from blood, and restore the proper balance of electrolytes in the blood. The patients with chronic kidney disease need three times a week for hemodialysis and may remove the inducers of betatrophin in blood. However, future studies are needed to elucidate this speculation.

## Conclusions

Taken together, our results show that circulating betatrophin concentrations were significantly increased in type 2 diabetic patients with different stages of albuminuria, in particular macroalbuminuric type 2 diabetic patients. Furthermore, betatrophin was correlated with duration of DM, SBP, BMI, FBG, HbA1C, ACR, and triglyceride, whereas it was inversely correlated with eGFR, and HDL-C. Dysregulated lipid metabolism may be the potential mechanism involved betatrophin in the development of DN. Thus, betatrophin may be a novel endocrine regulator involved in DN development. Further studies need to elucidate factors contributing to betatrophin regulation in humans as well as the pathophysiological significance of betatrophin upregulation in DN.

## Abbreviations

DN: Diabetic nephropathy
T1DM: Type 1 diabetes mellitus
T2DM: Type 2 diabetes mellitus
ACR: Albumin/creatinine ratio
ELISA: Enzyme-linked immunosorbent assay
SBP: Systolic blood pressure
BMI: Body mass index
eGFR: Estimated glomerular filtration rate
HDL-C: High-density lipoprotein cholesterol
LDL-C: Low-density lipoprotein cholesterol
OR: Odds ratio
ANOVA: Analysis of variance
FBG: Fasting blood glucose
HbA1c: Hemoglobin A1c
hs-CRP: High-sensitivity C-reactive protein
