# Erratum to: Higher serum betatrophin level in type 2 diabetes subjects is associated with urinary albumin excretion and renal function

**DOI:** 10.1186/s12933-017-0538-2

**Published:** 2017-04-26

**Authors:** Chang-Chiang Chen, Hendra Susanto, Wen-Han Chuang, Ta-Yu Liu, Arivajiagane Arundhathi, Chih-Hong Wang

**Affiliations:** 1 Department of Biological Science and Technology, National Chiao Tung University, 75 Bo-Ai Street, Hsinchu, 300 Taiwan; 2 Department of Internal Medicine, National Taiwan University Hospital Hsin-Chu Branch, Hsinchu, 300 Taiwan; 3 Institute of Molecular Medicine and Bioengineering, National Chiao Tung University, Hsinchu, Taiwan

## Erratum to: Cardiovasc Diabetol (2016) 15:3 DOI 10.1186/s12933-015-0326-9

After publication of the original article [1], it came to the authors’ attention that a contributor to the study, Arivajiagane Arundhathi, had been inadvertently omitted from the author list. Her affiliation, Institute of Molecular Medicine and Bioengineering, National Chiao Tung University, was also omitted.

The author list as appears in this erratum is correct. The “Competing interests” section does not require amending, and the “Authors’ contributions” section is corrected as follows:

- CHW conceived the research and contributed to the research project design, the data interpretation and writing of the manuscript. HS and CCC contributed to perform the experiments, participant recruiting, and clinical data compilation. CCC, HS, WHC, and TYL analyzed the data. AA and HS prepared blood samples for analysis. All authors read and approved the final manuscript.
